# Vitamin D deficiency, cardiothoracic ratio, and long-term mortality in hemodialysis patients

**DOI:** 10.1038/s41598-020-64359-9

**Published:** 2020-05-05

**Authors:** Heng-Jung Hsu, I-Wen Wu, Kuang-Hung Hsu, Chiao-Yin Sun, Chun-Yu Chen, Chin-Chan Lee

**Affiliations:** 1 Division of Nephrology, Chang Gung Memorial Hospital, Keelung, Taiwan; 2 College of Medicine, Chang Gung University, Tao-Yuan, Taiwan; 3 The Graduate Institute of Clinical Medical Sciences, Chang Gung University Medical College, Taoyuan School of Medicine, Taoyuan, Taiwan; 4 Community Medicine Research Center, Keelung Chang Gung Memorial Hospital, Keelung, Taiwan; 5 Healthy Aging Research Center, Chang Gung University, Taoyuan, Taiwan; 6 Laboratory for Epidemiology, Department of Health Care Management, Chang Gung University, Taoyuan, Taiwan; 7 Department of Emergency Medicine, Chang Gung Memorial Hospital, Taoyuan, Taiwan; 8 Department of Urology, Chang Gung Memorial Hospital, Taoyuan, Taiwan

**Keywords:** Cardiac hypertrophy, Haemodialysis

## Abstract

Hemodialysis patients are a special group of patients with higher mortality rates. Hemodialysis patients with vitamin D deficiency {plasma levels of 25-hydroxyvitamin D [25(OH)D] below 20 ng/mL} are associated with even higher mortality rates. The prognostic importance of vitamin D deficiency in hemodialysis patients with different cardiothoracic ratios (CTRs) is still unclear. This prospective study was performed in a single hemodialysis center, and 186 patients were included. This study analyzed the prognostic importance of vitamin D deficiency in hemodialysis patients with different CTRs. Vitamin D deficiency patients had a significantly higher prevalence of stroke and diabetic mellitus than those without vitamin D deficiency. In addition, the CTR was higher in patients with vitamin D deficiency than in those without vitamin D deficiency. After multivariate logistic regression, we found that CTR was the solitary factor that was independently significantly associated with vitamin D deficiency [odds ratio: 1.07, 95% confidence internal (CI): 1.01–1.13, *p* = 0.02]. Additionally, vitamin D deficiency was associated with all-cause mortality in patients with higher CTR after adjustment in hierarchical regression models. In conclusion, we reported that vitamin D deficiency was independently significantly associated with a higher CTR. We additionally revealed that vitamin D deficiency was an independent predicator for all-cause mortality in higher CTR hemodialysis patients.

## Introduction

Vitamin D deficiency {plasma levels of 25-hydroxyvitamin D [25(OH)D] below 20 ng/mL}is common in patients with chronic kidney disease (CKD) with or without dialysis. In CKD patients without dialysis, the prevalence of vitamin D deficiency is approximately 50~ 70%^1–4^. In CKD patients undergoing hemodialysis, the prevalence of vitamin D deficiency is approximately 50–98%^5–7^. The prevalence of vitamin D deficiency is even higher in CKD patients undergoing peritoneal dialysis, and the prevalence rates ranging from 86% to 100%^7,8^. The cause for the higher prevalence of vitamin D deficiency in CKD patients is not well-defined, but less exposure to sun, dietary restriction, and inefficient 25(OH)D formation are considered to be the possible causes^9^.

CKD patients are a special group of patients with higher mortality rates, including cardiovascular- and infection-related mortality^10^. In addition, diabetes mellitus (DM) is the primary cause of CKD that requires hemodialysis, accounting for nearly 50% of cases^11,12^. Vitamin D deficiency is linked with higher mortality risk in the general population^13–16^ and in CKD patients undergoing hemodialysis^17–19^. The possible reasons to explain the poor prognosis related with vitamin D deficiency are still unclear. However, some experimental studies with vitamin D receptor knock-out mice found impaired immunity, type 2 DM, and cardiovascular disease develop in those mice^20–22^, which indicates that the possible cause of the poor prognosis associated with vitamin D deficiency may be related to cardiovascular disease, infections, or comorbidity of type 2 DM. It is not surprising that CKD patients undergoing hemodialysis had higher all-cause mortality.

Several animal studies showed that vitamin D deficiency is connected with cardiac remodeling, including oxidative stress, fibrosis, apoptosis, cardiac hypertrophy, cardiac inflammation, left chamber alterations, and systolic dysfunction^23–25^. It seems that vitamin D is necessary for maintaining regular cardiac function. However, in clinical observational studies, it is difficult to define the complicated connection between vitamin D deficiency and prognosis in diabetes and hypertension hemodialysis patients. Patange *et al*. found that vitamin D deficiency is linked with diastolic dysfunction and left ventricular mass increase in children with CKD^26^. In addition, arterial wall stiffness was also found in children with vitamin D deficiency^27^. Cardiac disorders in pediatric CKD patients and vitamin D deficiency could provide information about the possible pathophysiology of vitamin D deficiency and mortality.

The cardiothoracic ratio (CTR) by a chest X-ray has been considered as a cardiac enlargement index, which indicates cardiac hypertrophy and volume overload^28^. The association between vitamin D deficiency and CTR in hemodialysis patients is still undefined. The prognostic importance of vitamin D deficiency in hemodialysis patients with cardiac hypertrophy and fluid overload is still uncertain. Therefore, we managed a cohort study to assess the relationship between CTR, vitamin D deficiency, and long-term mortality in hemodialysis patients with a follow-up period of approximate 5 years. The purpose of this present single dialysis center study was to evaluate the clinical characteristics of hemodialysis patients with vitamin D deficiency and to survey the predictive value of vitamin D deficiency on long-term prognosis in patients with different cardiac hypertrophy and fluid overload statuses.

## Results

### The characteristics and laboratory data of the study patients undergoing hemodialysis

**Figure 1:**
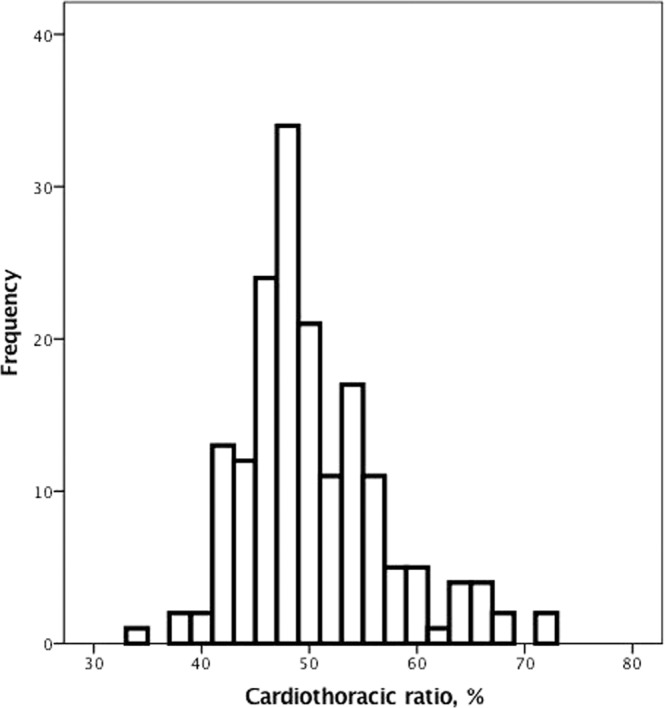
The distribution of the cardiothoracic ratio in our study patients with chronic kidney disease on hemodialysis.

**Figure 2:**
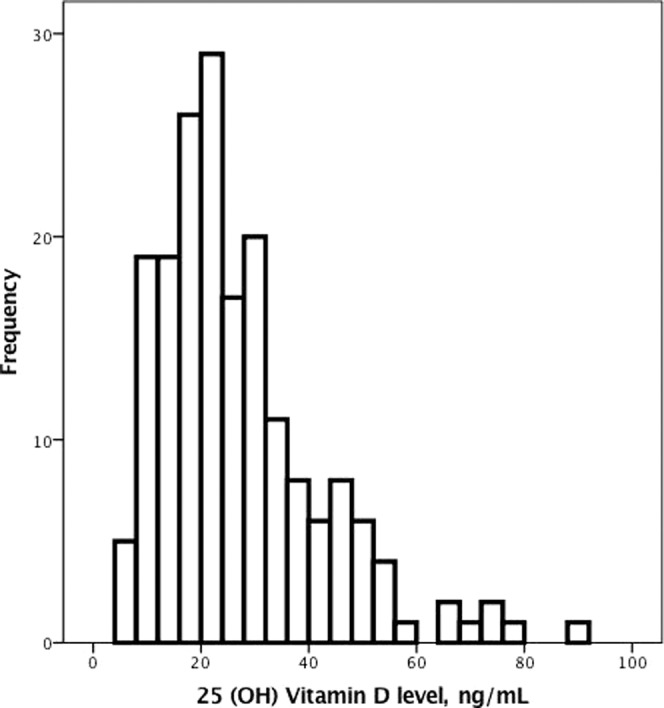
The distribution of serum 25 (OH) D levels among our study patients with chronic kidney disease on hemodialysis.

The mean age of our study dialysis patients was approximately 59 ± 14 years old (Table 1). The prevalence of DM was approximately 38%, and the average dialysis duration was approximately 7 ± 25 years. The nutritional status of the patients was adequate, with an albumin level of approximately 3.8 ± 0.4 g/dL and a normalized protein catabolic rate (nPCR) of approximately 1.2 ± 0.3 g/kg/day (Table 2). The CTR of the study patients was approximately 50 ± 7% (Fig. 1). The mean serum level of 25 (OH) D was approximately 27 ± 15 ng/mL (Fig. 2). The prevalence of vitamin D deficiency was approximately 37%.

**Table 1:** Baseline characteristics according to the presence or absence of vitamin D deficiency.

Variable	All patients	Normal	Vitamin D-deficient	*p* value
	N = 186	N = 117	N = 69	
Age (years)	59 ± 14	59 ± 12	59 ± 13	0.98
Male sex (%)	84 (45%)	54 (46%)	30 (44%)	0.72
Smoking (%)	29 (16%)	20 (18%)	9 (13%)	0.44
DM (%)	70 (38%)	38 (33%)	32 (48%)	0.04*
CAD (%)	33 (18%)	22 (19%)	11 (16%)	0.67
CHF (%)	39 (21%)	25 (22%)	14 (21%)	0.92
PAD (%)	22 (12%)	15 (13%)	7 (10%)	0.62
COPD (%)	14 (8%)	8 (7%)	6 (9%)	0.61
Peptic ulcer disease (%)	64 (34%)	39 (34%)	25 (37%)	0.61
Stroke (%)	19 (10%)	7 (6%)	12 (18%)	0.01*
Cancer (%)	15 (8%)	11 (10%)	4 (6%)	0.40
Dialysis duration (years)	7 ± 25	9 ± 31	4 ± 4	0.26
Body mass index (kg/m^2^)	23 ± 4	23 ± 4	23 ± 4	0.84
nPCR (g·kg^−1^·day^−1^)	1.2 ± 0.3	1.2 ± 0.3	1.1 ± 0.3	0.08
Cardiothoracic ratio (%)	50 ± 7	49 ± 6	52 ± 7	0.01*

Notes: Values are expressed as the mean ± SD or total number (percent).

*p value < 0.05.

Statistical significance based on Chi-square test for categorical variables or *t*-test for continuous variables.

Abbreviations: DM, diabetes mellitus; CAD, coronary artery disease; CHF, congestive heart failure; PAD, peripheral arterial occlusive disease; COPD, chronic obstructive pulmonary disease; nPCR, normalized protein catabolic rate.

**Table 2:** Biochemical and dialysis-related parameters according to the presence or absence of vitamin D deficiency.

Variable	All patients	Normal	Vitamin D-deficient	*p* value
	N = 186	N = 117	N = 69	
BUN (mg/dL)	70 ± 22	72 ± 24	67 ± 19	0.20
Creatinine (mg/dL)	11 ± 3	11 ± 3	10 ± 3	0.045*
Hemoglobin (g/dL)	10 ± 1	11 ± 1	10 ± 2	0.12
Albumin (g/dL)	3.8 ± 0.4	3.8 ± 0.4	3.7 ± 0.4	0.12
hs-CRP (mg/L)	10 ± 19	8 ± 14	10 ± 22	0.62
Calcium (mg/dL)	9 ± 1	9 ± 1	9 ± 1	0.86
Phosphate (mg/dL)	5.2 ± 1.8	5.1 ± 1.6	5.2 ± 1.7	0.80
Cholesterol (mg/dL)	180 ± 49	180 ± 50	170 ± 50	0.28
^$^Ca × P, mg^2^/dL^2^	49 ± 17	48 ± 17	49 ± 18	0.72
i-PTH (pg/mL)	360 ± 380	330 ± 330	450 ± 860	0.16
IL-1 beta (pg/mL)	1.8 ± 5.7	2.0 ± 7.4	1.5 ± 1.1	0.60
IL-6 (pg/mL)	6.5 ± 29.5	8.3 ± 38.8	3.9 ± 4.4	0.46
TNF-alpha (pg/mL)	17 ± 68	17 ± 65	19 ± 78	0.92
25(OH) Vitamin D (ng/mL)	27 ± 15	35 ± 14	14 ± 4	<0.001*
Kt/V	1.7 ± 0.4	1.7 ± 0.3	1.6 ± 0.4	0.17
URR (%)	0.7 ± 0.1	0.8 ± 0.1	0.7 ± 0.1	0.17

*p value <0.05.

Statistical significance based on *t*-test for continuous variables.

Abbreviations: BUN, blood urea nitrogen; hs-CRP, high-sensitivity C-reactive protein; i-PTH, intact parathyroid hormone; IL-1 beta, interleukin 1 beta; IL-6, interleukin 6; TNF-alpha, tumor necrosis factor-alpha; URR, urea reduction rate.

^$^Product of serum calcium and phosphate.

### Differences in characteristic and laboratory data between dialysis patients with and without vitamin D deficiency

**Figure 3:**
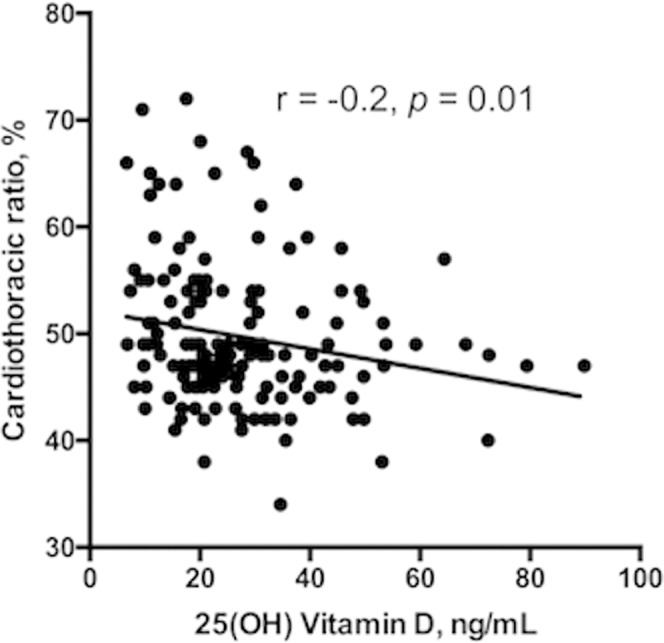
The correlation between serum 25 (OH) D levels and cardiothoracic ratio in our study patients with chronic kidney disease on hemodialysis.

Vitamin D deficiency patients had a significantly higher prevalence of DM (48% *vs*. 33%, *p* = 0.04) and stroke (18% *vs*. 6%, *p* = 0.01) than patients without vitamin D deficiency. The age, sex, smoking status and prevalence of congestive heart failure (CHF), coronary artery disease (CAD), chronic obstructive pulmonary disease (COPD), peripheral arterial occlusive disease (PAD), peptic ulcer disease, and cancer were similar between study patients with and without vitamin D deficiency. However, the CTR was greater in patients with vitamin D deficiency than in those without vitamin D deficiency (52 ± 7 *vs*. 49 ± 6, *p* = 0.01). The serum vitamin D levels were negatively associated with CTR (r = −0.2, p = 0.01) (Fig. 3).

Regarding levels of serum, vitamin D deficiency patients had significantly lower levels of creatinine (10 ± 3 *vs*. 11 ± 3 mg/dL, *p* = 0.045). Nutritional status was similar between study patients with and without vitamin D deficiency based on serum levels of albumin and nPCR. In addition, the calcium and phosphate products, the serum levels of calcium and phosphate were also not different in these two groups of patients. The serum levels of intact parathyroid hormone (iPTH) were higher in vitamin D deficiency patients than those without vitamin D deficiency 450 ± 860 *vs*. 330 ± 330 pg/mL, *p* = 0.16), but the difference was not significant. The levels of hemoglobin were also similar between these two groups of patients. The dialysis efficiency presented by the urea reduction rate and Kt/V were also similar between these two groups of patients. The inflammation markers, including interleukin (IL)−1 beta, IL-6, tumor necrosis factor (TNF)-alpha, and, high-sensitivity C-reactive protein (hs-CRP) were also not significantly different between the study patients with and without vitamin D deficiency. The serum levels of 25(OH)D were not unexpectedly lower in patients with vitamin D deficiency than in those without vitamin D deficiency (14 ± 4 *vs*. 35 ± 14, *p* < 0.001).

### Factors associated with 25 (OH) D deficiency

To identify the factors associated with vitamin D deficiency, we used univariate logistic regression and found that patients with poor fluid status or cardiac function who presented with high CTR, lower serum creatinine, and higher prevalence of DM and stroke were significantly more possible to develop vitamin D deficiency (Table 3). Then, we used multivariate logistic regression and found that the cardiothoracic ratio was independently significantly connected with vitamin D deficiency [odds ratio: 1.07, 95% confidence internal (CI): 1.01–1.13, *p* = 0.02].

**Table 3:** Factors associated with vitamin D deficiency in chronic kidney disease patients on hemodialysis.

Factor	Univariate odds ratio	95% confidence interval	*p*	Multivariate odds ratio	95% confidence interval	*p*
Age (years)	1.00	0.98–1.02	0.98	0.99	0.96–1.02	0.56
Male sex (%)	0.90	0.49–1.63	0.72	1.44	0.64–3.27	0.38
DM (%)	1.88	1.01–3.48	0.045*	1.26	0.60–2.63	0.54
Stroke (%)	3.40	1.27–9.12	0.02*	2.29	0.64–8.21	0.20
Cardiothoracic ratio (%)	1.07	1.01–1.12	0.01*	1.07	1.01–1.13	0.02*
Creatinine (mg/dL)	0.89	0.79–0.99	0.048*	0.89	0.76–1.05	0.16

Abbreviations: DM, diabetes mellitus.

*p value <0.05.

### Long-term prognosis between patients with and without vitamin D deficiency

**Figure 4:**
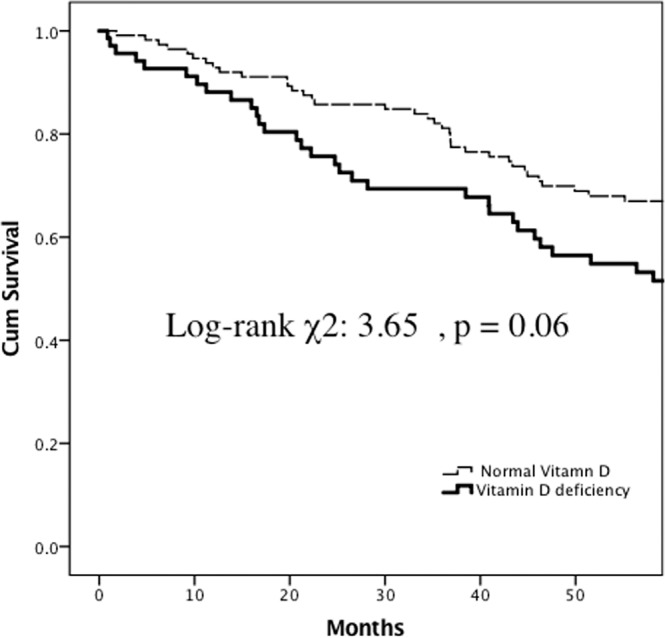
Five-year all-cause mortality-free Kaplan-Meier survival curves of chronic kidney disease patients on hemodialysis with 25(OH) D deficiency and normal 25 (OH) D levels (log-rank χ^2^: 3.65, p = 0.06).

In this 5-year follow-up study, we found that vitamin D deficiency hemodialysis patients had a trend of higher all-cause mortality than those without vitamin D deficiency (Fig. 4), although the difference was not significant (log-rank: χ^2^: 3.65, *p* = 0.06).

Because CTR was independently significantly associated with vitamin D deficiency, our study patients were sub-grouped by a CTR cutoff of 50%. Patients with a CTR higher than 50% were considered to have poor cardiac function and fluid status. In contrast, patients with a CTR less than 50% were considered to have good cardiac function and fluid status.

**Figure 5:**
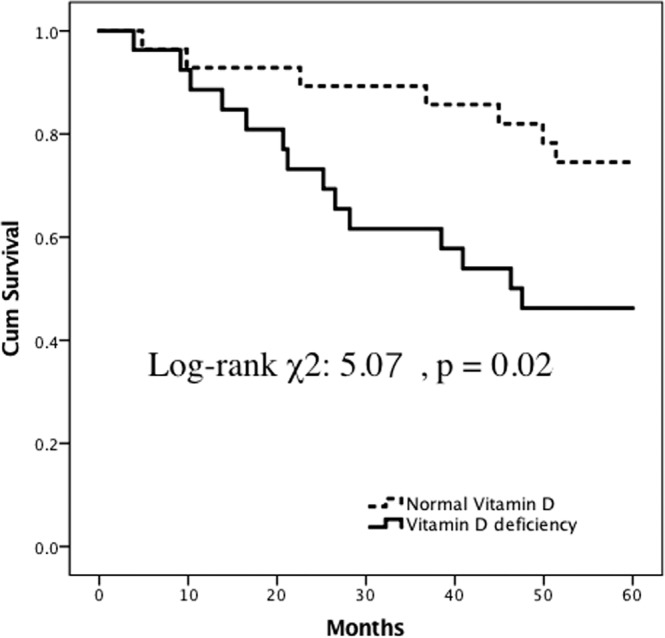
Five-year all-cause mortality-free Kaplan-Meier survival curves in hemodialysis patients with a high cardiac thoracic ratio (CTR) (CTR over 50%) between patients with 25(OH) D deficiency and normal 25 (OH) D levels (log-rank χ^2^: 5.07, p = 0.02).

**Figure 6:**
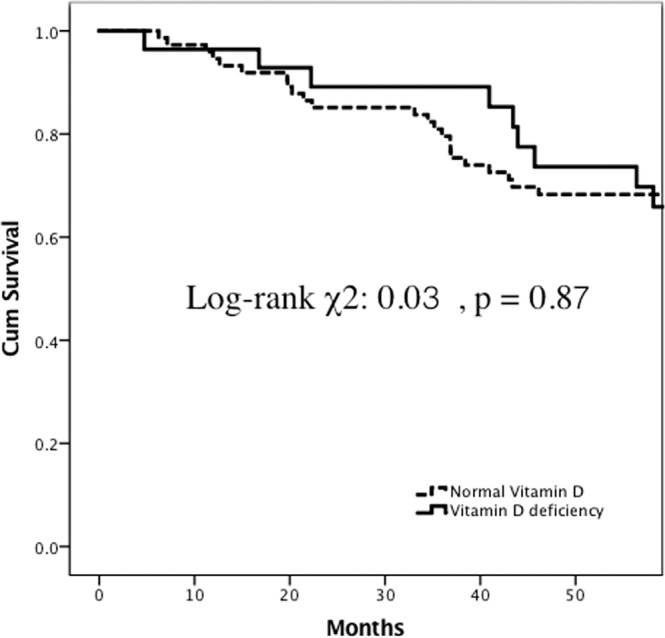
Five-year all-cause mortality-free Kaplan-Meier survival curves in hemodialysis patients with a low cardiac thoracic ratio (CTR) (CTR less than 50%) between individuals with 25(OH) D deficiency and normal 25 (OH) D levels (log-rank χ^2^: 0.03, p = 0.87).

In patients with poor fluid status and cardiac function, patients with vitamin D deficiency had significantly higher all-cause mortality than those without vitamin D deficiency (Fig. 5) (log-rank χ^2^: 5.07, *p* = 0.02). However, in patients with good fluid status and cardiac function, the all-cause mortality was similar between patients with and without vitamin D deficiency (Fig. 6) (log-rank χ^2^: 0.03, *p* = 0.87).

**Figure 7:**
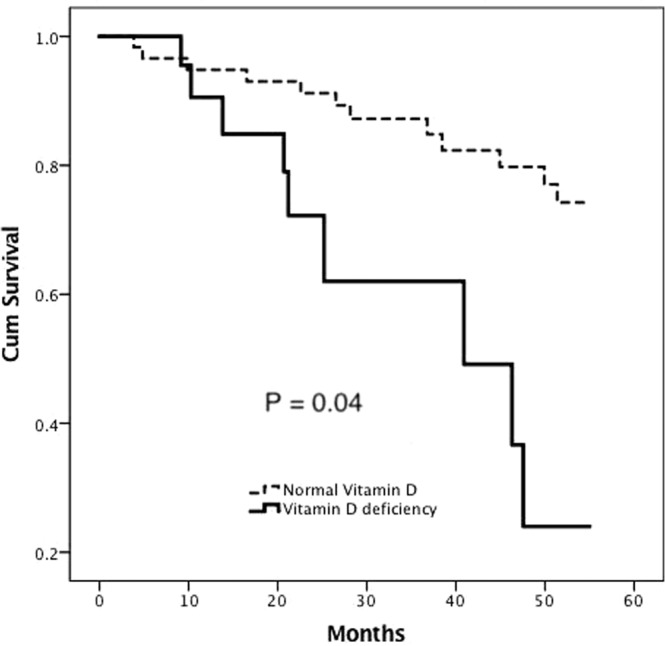
Five-year all-cause mortality-free Cox regression survival curves in hemodialysis patients with a high cardiac thoracic ratio (CTR) (CTR over 50%) between patients with 25(OH) D deficiency and normal 25 (OH) D levels (p = 0.04).

We used Cox regression to determine the prognostic value of vitamin D deficiency in patients with poor fluid status and cardiac function. Vitamin D deficiency was persistently significantly related with all-cause mortality in Model 1 (adjusted for age and sex) [Hazard ratio (HR): 2.5, 95% CI: 1.0–6.3, *p* = 0.048], Model 2 (HR: 2.6, 95% CI: 1.0–6.7, *p* = 0.04) (adjusted for comorbidities of DM, CHF, CAD), Model 3 (HR: 2.8, 95% CI: 1.1–7.1, *p* = 0.03) (adjusted for CTR), Model 4 (HR: 2.6, 95% CI: 1.0–6.5, *p* = 0.047) (adjusted for serum albumin), and Model 5 (HR: 3.1, 95% CI: 1.1–9.3, *p* = 0.04) (adjusted for hemoglobin, serum creatinine, hs-CRP, calcium, phosphate, and cholesterol) (Table 4) (Fig. 7).

**Table 4:** Multivariate Cox regression analysis for all-cause mortality in hemodialysis patients with poor fluid status.

Models	HR	95% CI	p value
**25 (OH) Vitamin D deficiency vs. normal 25 (OH) vitamin D: categorical variable**			
Unadjusted	2.7	1.1–6.8	0.03*
Model 1	2.5	1.0–6.3	0.048*
Model 2	2.6	1.0–6.7	0.04*
Model 3	2.8	1.1–7.1	0.03*
Model 4	2.6	1.0–6.5	0.047*
Model 5	3.1	1.1–9.3	0.04*

Model 1 was adjusted for age (1-year increment) and sex.

Model 2 was adjusted for comorbidities of diabetes mellitus, congestive heart failure and coronary artery disease.

Model 3 was adjusted for cardiothoracic ratio (%).

Model 4 was adjusted for serum albumin (1-g/dL increments).

Model 5 was adjusted for serum creatinine (1-mg/dL increments), hemoglobin (1-g/dL increments), high-sensitivity C-reactive protein (1-mg/L increments), calcium (1-mg/dL increments), phosphate (1-mg/dL increments) and cholesterol (1-mg/dL increments).

*p value <0.05.

Abbreviations: HR, hazard ratio; 95% CI, 95% confidence interval.

## Discussion

In this study, we found that hemodialysis patients with comorbidities of DM and stroke were significantly more possible to develop vitamin D deficiency. In addition, those patients with fluid overload or poor cardiac function evidenced by CTR were also significantly more likely to have vitamin D deficiency. In addition, vitamin D deficiency patients had significantly lesser serum creatinine levels than those without vitamin D deficiency. Vitamin D deficiency patients had a worse prognosis during this 5-year follow-up period, but the difference was not significant. However, vitamin D deficiency was significantly related with greater all-cause mortality in patients with higher CTR.

**Figure 8:**
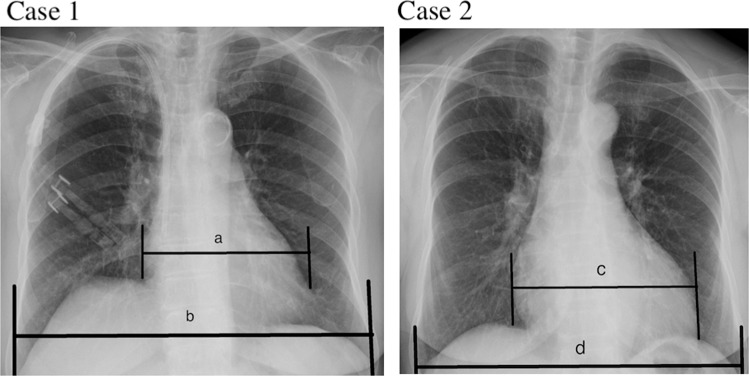
Examples of normal cardiothoracic ratio (CTR) (CTR less than 50%) and abnormal CTR (CTR over 50%) chest X-ray images. (In case 1, the CTR is a/b = 46%; in case 2, the CTR is c/d = 58%).

In this study, CTR was independently linked with vitamin D deficiency. CTR derived from chest X-ray is conventionally used to assess left ventricle (LV) size^29,30^ and is also associated with LV systolic dysfunction^31,32^ (Fig. 8). Furthermore, CTR is also used to determine fluid status in dialysis patients^28^. Vitamin D deficiency was related to various cardiovascular diseases, including hypertension^33^, increased carotid artery intima-medial thickness^34^, and cardiac hypertrophy^23^. The finding that vitamin D deficiency was significantly related with the CTR may be explained by the impaired cardiac function and remodeling linked with vitamin D deficiency. In additional, experimental studies similarly indicated that vitamin D deficiency was associated with cardiac inflammation, oxidative stress, energetic metabolic changes, fibrosis, apoptosis, cardiac hypertrophy, left chamber alterations, and systolic dysfunction^23^. Accordingly, this finding may further confirm the importance of vitamin D in the maintenance of normal cardiac function and repairing after stress or remodeling.

In this study by a follow-up duration of 5 years, a higher CTR was significantly related with poor prognosis. However, the prognostic value was less significant in all study patients. There are only few studies showing the predicative value of CTR for the long-term prognosis of hemodialysis patients. Chen *et al*. found that CTR was prognostic for all-cause mortality in 468 non-DM hemodialysis patients^35^, but Gao *et al*. showed that mortality risk was not associated by CTR^36^. In addition, Yujiro *et al*. also showed that CTR was not significantly connected with all-cause mortality except in those patients in the highest CTR quartile (0.535–0.569)^37^. Thus, the connection between CTR and all-cause mortality in hemodialysis patients has not been consistent between studies. Our study found that vitamin D deficiency was significantly associated with all-cause mortality in patients with a CTR higher than 0.5. Similar to the findings of the report by Yujiro *et al*., CTR and vitamin D deficiency were related with all-cause mortality. Heart dysfunction with hypertrophy, systolic dysfunction, and left chamber alterations were more prominent in patients with higher CTR. Vitamin D is likely essential for the maintenance of normal cardiac function and repair after stress or remodeling. Patients with higher CTR were considered to be the group of patients with poor fluid status and cardiac hypertrophy. Consequently, it is reasonable that vitamin D deficiency was connected with all-cause mortality in patients with higher CTR who suffering from more cardiac stress and fluid overload.

Comorbidity of DM was significantly associated with vitamin D deficiency in hemodialysis patients. Epidemiological and association study data clearly showed a correlation between vitamin D deficiency and a higher prevalence of DM^38^. Vitamin D deficiency provides to both the initial insulin resistance and the following onset of diabetes caused by beta-cell death. Vitamin D undertakes to reduce inflammation, which is a main component in the induction of insulin resistance^39^. Comorbidity of stroke was also significantly connected with vitamin D deficiency in hemodialysis patients. Due to the numerous non-calcemic autocrine and paracrine actions of vitamin D, an relationship between stroke and vitamin D deficiency has been stated, too. Judd *et al*. found that vitamin D deficiency was associated with incident stroke and the potency of this relationship does not show to differ by race^40^. Because vitamin D deficiency was also correlated with atrial fibrillation^41^, a higher prevalence of stroke in individuals with vitamin D deficiency may also excuse for atrial fibrillation. In addition, vitamin D levels are more strongly related to sun exposure. Patients with stroke might go outdoors less frequently due to their inability and have less sun exposure; therefore, it is not surprising that they are high risk for vitamin D deficiency.

We found that the inflammatory markers, comprising hs-CRP and inflammatory cytokines (IL-1 beta, IL-6, and TNF-alpha), were similar between hemodialysis patients with and without vitamin D deficiency. Numerous studies have reported that vitamin D is also essential for immune modulation through the immune cells vitamin D receptor^42^, and vitamin D deficiency was linked with death in critically ill patients^16,43,44^. Tiwari *et al*. reported significantly higher serum levels of IL-1 beta and IL-6 in vitamin D-deficient DM patients^45^. Kalkwarf *et al*. found that serum 25 (OH) D levels were inversely related with CRP and IL-6 in children and adolescents with CKD^4^. However, information on the relationship between inflammatory markers and vitamin D deficiency is requiring in CKD patients with hemodialysis. The results showed that the levels of TNF-alpha, IL-1 beta, IL-6, and hs-CRP were not connected with vitamin D deficiency. Furthermore, vitamin D deficiency was not linked with infection-related mortality (data not shown). These findings may provide information about vitamin D deficiency, inflammation, and infection-related death in hemodialysis patients.

There are several limitations to this study. First, we do not get records on the left ventricular mass from echocardiography, the gold standard for the evaluation of cardiac hypertrophy. Therefore, we cannot discuss whether CTR indicates fluid overload or cardiac hypertrophy. Second, the study results were built on one measurements of the variables, including the CTR and serum 25(OH) D levels. Therefore, the results might overestimate or underestimate the true association. In addition, seasonal variation in 25 (OH) D levels in hemodialysis patients has also been observed, with lower levels in winter and higher levels in summer. However, we checked the serum levels of vitamin D from dialysis patients within almost the same month (no more than 2 months). Therefore, the seasonal variation in 25 (OH) D levels might not be prominent in our study. Fourth, the results of this observational study do not certainly show causation. Therefore, the study does not provide the goal range of CTR in clinical practice. Fifth, because this is a cohort study that enrolled approximately 186 patients in a dialysis center with approximately 435 dialysis patients in total in Taiwan, our results need to be confirmed by more studies with a large sample size. In addition, other types of vitamin D, such as 1,25-di-OH-D or vitamin D binding protein, were not measured. However, our results revealed an independent significant connection between CTR and vitamin D deficiency and revealed the prognostic value of vitamin D deficiency in higher CTR patients. These findings may emphasize the importance of vitamin D in maintaining cardiac function and morphology and improving long-term outcomes in excess fluid overload or cardiac hypertrophy patients. In addition, this finding may raise further questions concerning vitamin D supplementation in hemodialysis patients with higher CTR to reduce all-cause mortality.

## Conclusion

We showed that vitamin D deficiency was independently significantly related with higher CTR. We additionally revealed that vitamin D deficiency was an independent predicator of all-cause mortality in hemodialysis patients with higher CTR. Further studies are needed to approve these consequences.

## Materials and Methods

### Study population and design

This was a prospective cohort study in single dialysis center that was accomplished to analyze the factors associated with vitamin D deficiency and the prognostic value of vitamin D on the mortality of hemodialysis patients with different statuses of cardiac hypertrophy and fluid overload. The total of 186 patients who had been undertaking regular dialysis for more than 6 months were joined. Those patients were excluded from the study if they were under 20 years old, were hospitalized for acute illnesses, or had not proposed their informed consent for participation. The patients were separated into 2 groups according to their levels of plasma 25 (OH) D by increments of 20 ng/mL^46–49^. Patients with plasma levels of 25 (OH) D below 20 ng/mL were thought to be vitamin D deficient. The study period was from August 1, 2006 to July 31, 2011. Patients who were absent from our dialysis program, shifted to peritoneal dialysis, or experienced transplantation were censored. Overall mortality was analyzed and compared between groups with vitamin D deficiency and without vitamin D deficiency. This study was approved by Chang Gung Memorial Hospital the Institutional Review Board (IRB) of the (99–2112B). Besides, all processes were performed in accordance with the related regulations and guidelines.

We collected basic demographic data for each patient, including age, sex, nutritional status (assessed by body mass index and nPCR), dialysis duration, and the presence/absence of DM, CAD, CHF, PAD, COPD, peptic ulcer disease, stroke, and cancer. We additionally collected the baseline biochemical, hematological, and dialysis-related parameters of these patients. DM was defined by the American Diabetes Association criteria in 1997. The utilization of more than 10 cigarettes per day for at least 1 year was considered as smoking. CHF was diagnosed by the Framingham criteria for heart failure. CAD was diagnosed by coronary angiography, PAD was diagnosed by peripheral arterial Doppler examination or amputation history, COPD was identified by pulmonary function tests and chest radiography, peptic ulcer disease was diagnosed by panendoscopy result, stroke was diagnosed by imaging studies and clinical symptoms, and cancer was diagnosed by pathological studies.

### Cardiothoracic ratio evaluation

Posterior-anterior chest radiograph was taken to measure the CTR just after hemodialysis sessions for all study patients at our center. CTR was expressed as the ratio of the width of the heart to the thoracic width by a posterior to anterior view chest X-ray about 120 kV acquired in a standing position with inspiration entirely. The thoracic width was calculated as the greatest diameter of the inner borders of the ribs and the cardiac width was calculated as the sum of the right and left greatest diameters from the midline. The CTR was analyzed by dividing the cardiac width by the thoracic width. Therefore, a CTR > 0.5 was defined as cardiomegaly, and higher CTR levels reveal a greater severity of cardiomegaly or fluid overload.

### Laboratory studies

We got whole laboratory data and dialysis-related parameters for patients in both groups. The laboratory profiles included the hemoglobin, plasma levels of albumin, blood urea nitrogen (BUN), creatinine, hs-CRP, calcium, phosphate, i-PTH, and cholesterol. Besides, parameters reflecting urea clearance, including Kt/V value (Daugirdas) and URR were evaluated to determine the dialysis treatment adequacy. Blood samples were collected and performed according to the recommended procedures by the National Kidney Foundation Kidney Disease Outcomes Quality Initiative guidelines. Plasma levels of hemoglobin, BUN, creatinine, hs-CRP, albumin, cholesterol, calcium, and phosphate were determined by spectrophotometric analysis by using the modified kinetic Jaffe reaction. Plasma i-PTH levels were evaluated by a commercially available radioimmunoassay kit (Scantibodies Laboratory; Santee, CA, USA). 25-OH vitamin D was measured at the initial assessment by electrochemiluminescence immunoassay (Cobas® Vitamin D3 assay by Roche Diagnostics GmbH, Mannheim, Germany) with an inter-assay coefficient of variation of 2.2–13.6% from blood samples drawn before the beginning of dialysis session.

### Statistical analysis

Group difference in categorical variables was determined via either the chi-square test or Fisher’s exact test. Continuous variables are presented as the means ± standard deviations. A two-tailed Student’s unpaired t test was employed to evaluate the differences between the means for those normally distributed continuous variables. To evaluate the factors linked with vitamin D deficiency in the hemodialysis patients, univariate followed by multivariate logistic regression models were applied. Mortality was treated as the end point in the survival analysis for all-cause mortality. Besides, patient survival was evaluated using the Cox proportional hazards model to control for covariates. We used Cox proportional hazards regression models to compare survival between groups with and without vitamin D deficiency. Hierarchical regression models were engaged to construct a potent covariate set for adjustment in the major hypothesized variable of vitamin D deficiency. A p value <0.05 was cogitated statistically significant. All analyses were made by means of commercially available statistics software SPSS software version 23.0 (SPSS; Chicago, IL, USA) for Mac.

### Ethics approval and consent to participate

This study was approved by the Institutional Review Board (IRB) of the Chang Gung Memorial Hospital (99–2112B), and informed consent was obtained from all participants of the study.

## Data Availability

All data generated or analyzed during this study are included in this published article.

## References

[1] Jabbar Z (2009). High prevalence of vitamin D deficiency in north Indian adults is exacerbated in those with chronic kidney disease. Nephrology.

[2] Patel NM (2010). Vitamin D deficiency and anemia in early chronic kidney disease. Kidney Int..

[3] Echida Y, Mochizuki T, Uchida K, Tsuchiya K, Nitta K (2012). Risk factors for vitamin D deficiency in patients with chronic kidney disease. Intern. Med..

[4] Kalkwarf HJ (2012). Vitamin D deficiency is common in children and adolescents with chronic kidney disease. Kidney Int..

[5] Bhan I, Burnett-Bowie SA, Ye J, Tonelli M, Thadhani R (2010). Clinical measures identify vitamin D deficiency in dialysis. Clin. J. Am. Soc. Nephrol..

[6] Wolf M (2007). Vitamin D levels and early mortality among incident hemodialysis patients. Kidney Int..

[7] Drechsler C (2011). Vitamin D status and clinical outcomes in incident dialysis patients: results from the NECOSAD study. Nephrol. Dial. Transpl..

[8] Anand S (2011). Vitamin D deficiency, self-reported physical activity and health-related quality of life: the Comprehensive Dialysis Study. Nephrol. Dial. Transpl..

[9] Jacob AI, Sallman A, Santiz Z, Hollis BW (1984). Defective photoproduction of cholecalciferol in normal and uremic humans. J. Nutr..

[10] Lee CC, Sun CY, Wu MS (2009). Long-term modality-related mortality analysis in incident dialysis patients. Perit. Dial. Int..

[11] Collins AJ (2014). US Renal Data System 2013 Annual Data Report. Am. J. Kidney Dis..

[12] Hill CJ, Fogarty DG (2012). Changing trends in end-stage renal disease due to diabetes in the United kingdom. J. Ren. Care.

[13] Zittermann A (2012). Vitamin D deficiency and mortality risk in the general population: a meta-analysis of prospective cohort studies. Am. J. Clin. Nutr..

[14] Arnson Y, Gringauz I, Itzhaky D, Amital H (2012). Vitamin D deficiency is associated with poor outcomes and increased mortality in severely ill patients. QJM.

[15] Venkatram S (2011). Vitamin D deficiency is associated with mortality in the medical intensive care unit. Crit. Care.

[16] de Haan K, Groeneveld AB, de Geus HR, Egal M, Struijs A (2014). Vitamin D deficiency as a risk factor for infection, sepsis and mortality in the critically ill: systematic review and meta-analysis. Crit. Care.

[17] Drechsler C (2010). Vitamin D deficiency is associated with sudden cardiac death, combined cardiovascular events, and mortality in haemodialysis patients. Eur. Heart J..

[18] Walker JP (2014). Vitamin D deficiency is associated with mortality and adverse vascular access outcomes in patients with end-stage renal disease. J. Vasc. Surg..

[19] Anand S (2013). Vitamin D deficiency and mortality in patients receiving dialysis: the Comprehensive Dialysis Study. J. Ren. Nutr..

[20] Bouillon R (2008). Vitamin D and human health: lessons from vitamin D receptor null mice. Endocr. Rev..

[21] O’Kelly J (2002). Normal myelopoiesis but abnormal T lymphocyte responses in vitamin D receptor knockout mice. J. Clin. Invest..

[22] Zeitz U (2003). Impaired insulin secretory capacity in mice lacking a functional vitamin D receptor. FASEB J..

[23] Assalin HB (2013). Impact of the length of vitamin D deficiency on cardiac remodeling. Circ. Heart Fail..

[24] Gupta GK, Agrawal T, DelCore MG, Mohiuddin SM, Agrawal DK (2012). Vitamin D deficiency induces cardiac hypertrophy and inflammation in epicardial adipose tissue in hypercholesterolemic swine. Exp. Mol. Pathol..

[25] Gezmish O (2010). Maternal vitamin D deficiency leads to cardiac hypertrophy in rat offspring. Reprod. Sci..

[26] Patange AR, Valentini RP, Gothe MP, Du W, Pettersen MD (2013). Vitamin D deficiency is associated with increased left ventricular mass and diastolic dysfunction in children with chronic kidney disease. Pediatr. Cardiol..

[27] Patange AR, Valentini RP, Du W, Pettersen MD (2012). Vitamin D deficiency and arterial wall stiffness in children with chronic kidney disease. Pediatr. Cardiol..

[28] Harasawa H, Yamazaki C, Itoh A, Masuko K (1989). [Cardiothoracic ratio and roentgenologic heart size as the indices of body fluid retention in uremics under hemodialysis]. Nihon Igaku Hoshasen Gakkai Zasshi.

[29] Laczkovics A (1988). Noninvasive assessment of acute rejection after orthotopic heart transplantation: value of changes in cardiac volume and cardiothoracic ratio. J. Cardiovasc. Surg..

[30] Hammermeister KE, Chikos PM, Fisher L, Dodge HT (1979). Relationship of cardiothoracic ratio and plain film heart volume to late survival. Circulation.

[31] Cohn JN (1993). Ejection fraction, peak exercise oxygen consumption, cardiothoracic ratio, ventricular arrhythmias, and plasma norepinephrine as determinants of prognosis in heart failure. The V-HeFT VA Cooperative Studies Group. Circulation.

[32] Bertolet BD (1991). Unrecognized left ventricular dysfunction in an apparently healthy alcohol abuse population. Drug. Alcohol. Depend..

[33] Martins D (2007). Prevalence of cardiovascular risk factors and the serum levels of 25-hydroxyvitamin D in the United States: data from the Third National Health and Nutrition Examination Survey. Arch. Intern. Med..

[34] Targher G (2006). Serum 25-hydroxyvitamin D3 concentrations and carotid artery intima-media thickness among type 2 diabetic patients. Clin. Endocrinol..

[35] Chen KH (2008). Cardiothoracic ratio, malnutrition, inflammation, and two-year mortality in non-diabetic patients on maintenance hemodialysis. Kidney Blood Press. Res..

[36] Gao N (2011). Measurements on the routine chest radiograph as prognostic markers in Chinese peritoneal dialysis patients. Clin. Nephrol..

[37] Okute Y (2017). Cardiothoracic Ratio as a Predictor of Cardiovascular Events in a Cohort of Hemodialysis Patients. J. Atheroscler. Thromb..

[38] Mathieu C (2015). Vitamin D and diabetes: Where do we stand?. Diabetes Res. Clin. Pract..

[39] Berridge MJ (2017). Vitamin D deficiency and diabetes. Biochem. J..

[40] Judd SE (2016). Vitamin D deficiency and incident stroke risk in community-living black and white adults. Int. J. Stroke.

[41] Thompson J, Nitiahpapand R, Bhatti P, Kourliouros A (2015). Vitamin D deficiency and atrial fibrillation. Int. J. Cardiol..

[42] Schwalfenberg GK (2011). A review of the critical role of vitamin D in the functioning of the immune system and the clinical implications of vitamin D deficiency. Mol. Nutr. Food Res..

[43] Zhang YP, Wan YD, Sun TW, Kan QC, Wang LX (2014). Association between vitamin D deficiency and mortality in critically ill adult patients: a meta-analysis of cohort studies. Crit. Care.

[44] Zajic P, Amrein K (2014). Vitamin D deficiency in the ICU: a systematic review. Minerva Endocrinol..

[45] Tiwari S, Pratyush DD, Gupta SK, Singh SK (2014). Vitamin D deficiency is associated with inflammatory cytokine concentrations in patients with diabetic foot infection. Br. J. Nutr..

[46] Forrest KY, Stuhldreher WL (2011). Prevalence and correlates of vitamin D deficiency in US adults. Nutr. Res..

[47] Holick MF (2011). Evaluation, treatment, and prevention of vitamin D deficiency: an Endocrine Society clinical practice guideline. J. Clin. Endocrinol. Metab..

[48] Oh TR (2017). Association between vitamin D deficiency and health-related quality of life in patients with chronic kidney disease from the KNOW-CKD study. PLoS One.

[49] Kara, A. V., Aldemir, M. N., Soylu, Y. E. & Arslan, Y. K. Relationship between Serum Vitamin D Levels and Health-Related Quality of Life in Maintenance Hemodialysis Patients. *Blood Purif*, 1-9, 10.1159/000497242 (2019). 10.1159/000497242 30799428

